# Funds of the Past, Present, and Future

**Published:** 1918-07-13

**Authors:** 


### WAR CHARITY AND RELIEF.

### Funds of the Past, Present, and Future.

The annual report of the Royal Patriotic Fund Corporation, an institution which represents a group of public relief funds of the past, is suggestive of the far-reaching and long-enduring effects of an international calamity, such as a great war. Hardly anybody now alive will be living when the last direct sufferer by the great European war will cease to receive, through State or charitable funds, relief in the form of pension or grant. The Civil War in America terminated in 1865; since then over nine hundred million pounds have been paid by the United States Treasury to pensioners, and the annual sum now dispensed to surviving soldiers, widows, and dependants is still an important item in the Budget. Our Minister of Pensions, in a recent report, has given us an indication of our own colossal commitments in respect of the war, but even he could not venture an estimate of the final obligations of the Empire in this connection.

Of course, State payment and charitable payment are different things, but they necessarily must be associated at times, and the second will be governed in its extent, as it has no doubt largely been in the past, by the generosity or niggardliness of the first. Charitable effort reaches to a sphere regulated by what current public opinion considers is beyond State obligation. If the public opinion of bygone days had been the same as that of to-day some of the funds now administered by the Royal Patriotic Fund Corporation need never have been started. But, apart from this reflection, their present working is a striking illustration of the aftermath of war. The report for the year 1916 shows that the Corporation relieved in twelve months 170,701 persons who were still adversely affected by the wars and calamities of the past. Some hundreds of those persons were widows and other dependants of men who served in the Russian war of 1854 to 1856, and fifty-six were similarly representative of officers and men who lost their lives through the foundering of King's ships in 1870, 1878, and 1881. The Indian Mutiny, that far-off event, and several minor forgotten campaigns also provided recipients of relief.

### Huge Capital of the Relief Fund.

These funds of the past, then, have not been too precipitate in operation. Perhaps it is the feeling that the probable economic conditions of coming years are still undiscovered that causes the Committee of the National Relief Fund to rest on their oars for the moment and to conserve their resources. The financial position of the Fund may, roughly, be stated to be one in which the payments are covered by the interest on investments and bank deposits, the capital thus represented amounting to £2,779,546. Nobody can say when and how this very respectable reserve will be expended. At present, with the exception of the unavoidable distress in East Coast towns, there is little call for extensive relief. Even the professional classes, who were as badly hit as anyone in the first year of the war, drew only £7,566 from the Fund in the six months ending September 30, 1917, as compared with £10,864 in the preceding period.

The Charity Commissioners, in their sixty-fifth report, have something to say about the smaller war charities, and deal with the work and records of the Commission in relation to charity generally. In 1917 the aggregate amount given by will for charitable purposes without any requirement for permanent investment was £1,594,845, the figures including valuation of residuary estates so far as it has been found practicable to calculate them. There were 347 permanent charitable bequests and devises, as compared with 280 during the preceding year. These figures have, perhaps, little to do with war charities, but are useful as indicating the fluid state of charitable revenue during a great war, a condition which has recently been observed as characterising also the gifts of the living. The report of the Commissioners refers at some length to the duties devolving upon them under the War Charities Act, 1916. The wasteful method of raising money through Flag-day collections, as it can be described in some instances, is sharply commented upon, and there is a suggestion of regret that the powers of registration authorities do not sufficiently safeguard the public in this regard.

### Figures of Flag-Day Collections.

Since the publication of this criticism, the Commissioner of Police has issued a statement setting forth his experiences in this regard. The statistics compiled from accounts presented of street collections in the metropolitan area between May 13, 1916, and April 9, 1918, show that a total sum of £286,830 has been collected at a cost of £51,432, leaving a net amount of £235,398 applicable to the objects for which the collections were made. The average cost of collection is thus shown to be rather under 18 per cent., inclusive of charges for organisation, advertisement, and the provision of collection boxes, flags and other emblems. The opinion of the Commissioner is that the percentage is no doubt high, but not so high as in itself to justify any sweeping condemnation of the system. Where the expenditure is shown in the accounts to be unduly large, attention is drawn to the fact, and, in the event of a renewed application, a reduction is insisted upon. It is reassuring to know that the police authorities can under present powers keep a tight hand on any possible leakage or abuse. At the best the Flag Day can be regarded only as an emergency method of raising money; but while it lasts it is as well that its regulation should be in the hands of competent people, who will carefully consider all its devolopments.

Charity, like most things, is affected by economic conditions, in the coming years a quantity liable to acute variations. A student of these conditions will find useful the list of Commissions and Committees set up to deal with questions which will arise at the close of the war, recently published by H.M. Stationery Office. The references of these Commissions and Committees cover a wide range of subjects, including development of trade, finance, supply of raw materials, coal and power, intelligence, scientific and industrial research, demobilisation and disposal of stores, labour and employment, agriculture and forestry, public administration, housing, education, treatment of aliens, and legal problems. The findings will no doubt lead to legislation of an important character. Legislation thus in embryo, that recently passed, and that immediately in preparation will change the lives of millions. If it is uncomfortable to live in unsettled days, none can gainsay that it is an interesting privilege to be alive to see a new England and a new world in the making.

